# The Diagnostic Ability of Ganglion Cell Complex Thickness-to-Total Retinal Thickness Ratio in Glaucoma in a Caucasian Population

**DOI:** 10.4274/tjo.galenos.2019.19577

**Published:** 2020-03-05

**Authors:** Almila Sarıgül Sezenöz, Sirel Gür Güngör, Ahmet Akman, Caner Öztürk, Şefik Cezairlioğlu, Mustafa Aksoy, Meriç Çolak

**Affiliations:** 1 Başkent University Hospital, Clinic of Ophthalmology, Ankara, Turkey; 2 Başkent University Health Sciences Faculty, Department of Ophthalmology Ankara, Turkey

**Keywords:** Ganglion cell complex, glaucoma, optical coherence tomography

## Abstract

**Objectives:**

To evaluate the diagnostic accuracy of the macular ganglion cell complex-to-total retinal thickness (G/T) ratio in a Caucasian population.

**Materials and Methods:**

A total of 86 patients were enrolled in this cross-sectional study. Patients were divided into 4 groups: healthy; ocular hypertension; preperimetric glaucoma; and early glaucoma. Macular ganglion cell complex (mGCC) thickness, total retinal thickness, and retinal nerve fiber layer thickness (RNFLT) in one randomly selected eye of each patient were measured with measured with Heidelberg HD spectral domain optical coherence tomography (Heidelberg Engineering, Inc., Heidelberg, Germany). G/T ratio (%) was calculated as (mGCC thickness / total retinal thickness) ×100. The ability of each parameter to diagnose glaucoma was examined by area under the receiver operating characteristic curve (AUROC) analysis and sensitivity evaluation at a fixed level of specificity. Unpaired t test was used to compare the measured values between the healthy subjects and the different patient groups.

**Results:**

The study included 9 healthy individuals, 18 patients with ocular hypertension, 28 with preperimetric glaucoma, and 31 with early glaucoma. Total retinal thickness, mGCC thickness, RNFLT, and G/T ratio were highest in the healthy group and decreased progressively in patients with ocular hypertension, preperimetric glaucoma, and early glaucoma. All comparisons between the groups were significant for these parameters (p<0.001 for all). Average RNFLT, average GCC, and total retinal thickness showed consistently higher AUROC than G/T ratio in the differentiation between healthy individuals and patients with ocular hypertension, preperimetric glaucoma, and early glaucoma.

**Conclusion:**

G/T ratio does not contribute to separation of ocular hypertension, preperimetric glaucoma, and early glaucoma patients from the healthy population. Compared to the other parameters investigated, G/T had lower diagnostic value.

## Introduction

Glaucoma is a progressive optic neuropathy characterized by retinal ganglion cell loss and thinning of the retinal nerve fiber layer (RNFL).^1,2^ Glaucomatous visual field defects emerge after 30% of the retinal ganglion cells are lost. Therefore, structural tests are important in the early diagnosis and follow-up of glaucoma.^3^

Previous optical coherence tomography (OCT) studies demonstrated peripapillary RNFL thinning in early glaucoma.^4,5,6^ More recent studies have shown that in addition to the peripapillary RNFL, changes in inner macular thickness parameters also occur related to glaucomatous ganglion cell loss.^7,8,9,10,11,12,13,14^ The total of the macular nerve fibers, ganglion cells, and inner plexiform layer is called the macular ganglion cell complex (mGCC).^15^ Current OCT devices can obtain mGCC measurements automatically, and studies report that a decrease in mGCC thickness has high diagnostic value in early glaucoma, similar to RNFL parameters.^11,15,16,17,18,19,20,21,22,23^

Reported mGCC thickness values range between 76.6 and 119.8 µm in normal eyes and between 53.6 and 99.1 µm in perimetric glaucomatous eyes.^16^ Because the range of mGCC thickness overlaps in normal and glaucomatous eyes, Kita et al.^15^ compared various macular parameters in Japanese patients and demonstrated that the ratio of mGCC thickness to total retinal thickness (G/T ratio) was the parameter with the highest diagnostic value in glaucoma.However, in a later study of white Europeans, G/T ratio was found to have a lower area under the receiver operating characteristic curve (AUROC) value than RNFL.^24^

The aim of this study was to determine the diagnostic value of G/T ratio for glaucoma in a Caucasian patient population.

## Materials and Methods

This study was approved by the Başkent University Ethics Committee. Data from a total of 130 patients who presented to the glaucoma unit of the ophthalmology outpatient clinic of Başkent University Hospital between December 2017 and June 2019 were reviewed for inclusion in this cross-sectional study. All patients underwent ophthalmologic examinations and their medical histories were analyzed. Ophthalmologic examination included visual acuity, slit-lamp anterior segment examination, gonioscopy, intraocular pressure measurements with Goldmann applanation tonometry, and dilated fundus examination. The results of 24-2 visual field tests performed with a Humphrey standard automated perimeter (Humphrey-Zeiss Systems, Dublin, CA) were evaluated. Tests with less than 20% fixation loss and false positive and false-negative rates both below 33% were regarded as reliable.

A Heidelberg HD spectral domain OCT (Heidelberg Engineering, Inc., Heidelberg, Germany) device was used to evaluate the optic nerve head and macular parameters of the patients. Measurements were made after pupil dilation by the same experienced technician at the same time of day. Patients whose images had signal strength values below 6 were not included in the study. Macular thickness was measured using an automated system. G/T ratio (%) was calculated using the formula (mGCC thickness / total retinal thickness) ×100.

The patients were divided into 4 groups for evaluation: healthy, ocular hypertension, preperimetric glaucoma, and early glaucoma. Those included in the healthy group had intraocular pressure below 21 mmHg, normal optic nerve appearance, normal anterior chamber angle, and normal visual field test results. The criteria for diagnosing open-angle glaucoma in the study patients were: open iridocorneal angle on gonioscopic examination, glaucomatous optic nerve damage (focal thinning or notching of the neuroretinal rim or diffuse thinning of the neuroretinal rim), and glaucomatous visual field defect in the absence of any other ocular disease that could be linked to visual field defect. Glaucomatous visual field defect was defined as meeting at least two of the following three criteria: (1) glaucoma hemifield test results outside normal limits; (2) presence of three locations with P <5% of normal distribution and one location with p<1% on pattern deviation plot; and (3) pattern standard deviation value with p<5%. These visual field defects were confirmed by two consecutive reliable visual field tests (fixation loss ≤20%, false positive and false negative error rates ≤25%). The individuals’ fellow eyes had open angle on gonioscopy and normal disc appearance and visual field results. Glaucomatous eyes with a mean deviation (MD) value of ≤-6 dB upon enrollment were grouped as early glaucoma. Patients with glaucomatous optic nerve damage but normal visual field were classified as preperimetric glaucoma. Patients with a normal optic disc and visual field but intraocular pressure above 21 mmHg were included in the ocular hypertension group.

Criteria for inclusion in the study were sufficient central vision for optimal fixation, adequate image quality for optimal evaluation, and no macular pathology on stereoscopic evaluation of the study eye.

### Statistical Analysis

Statistical analyses were performed using IBM SPSS 21 statistics software (SPSS Inc., Chicago, IL). The adequacy of each parameter for diagnosing glaucoma was determined through AUROC analysis and evaluating sensitivity at a fixed level of specificity. Measured values of the healthy individuals and selected patient groups were compared using ANOVA.

## Results

Ninety-five eyes of 95 patients were included in the study. Data from 18 healthy subjects, 18 patients with ocular hypertension, 28 patients with preperimetric glaucoma, and 31 patients with early glaucoma were analyzed. Comparisons of age, sex, and refraction values of the 95 study patients revealed no significant differences between the groups (p≥0.05). The patients’ clinical characteristics are shown in Table 1.

It was observed that mGCC thickness, total retinal thickness, RNFL thickness, and G/T ratio were highest in healthy subjects and decreased respectively in patients with ocular hypertension, patients with preperimetric glaucoma, and patients with early glaucoma. All between-group comparisons based on these values were statistically significant (p<0.001 for all) (Table 2).

**Figure 1:**
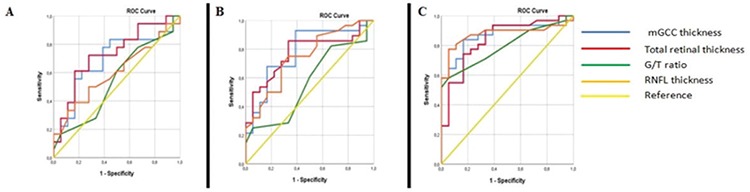
Receiving operator characteristic curves for mGCC thickness, total retinal thickness, RNFL thickness, and G/T ratio. A) Healthy vs. ocular hypertension; B) Healthy vs. preperimetric glaucoma; C) Healthy vs. early glaucoma

For distinguishing healthy subjects from early glaucoma patients, AUROC values for mean mGCC, RNFL thickness, total retinal thickness, and G/T ratio were 0.876, 0.876, 0.840, and 0.794, respectively. Similarly, AUROC values were higher for mean mGCC, RNFL thickness, and total retinal thickness values compared to G/T ratio in the differentiation between healthy subjects and patients with ocular hypertension and preperimetric glaucoma (Table 3). ROC values for all parameters are shown in Figure 1A, B, and C.

## Discussion

In the present study, we evaluated the diagnostic power of mGCC thickness, RNFL thickness, total retinal thickness, and G/T ratio for glaucoma in our patients.

The diagnostic power of macular thickness parameters has been previously investigated in OCT studies, but it was shown that total macular thickness values were not as valuable as RNFL in the diagnosis of glaucoma.^6,11,12,25^ When subsequently developed macular segmentation algorithms were used to separately evaluate the thicknesses of the macular nerve fiber and inner retinal layers, values comparable to RNFL were obtained.^14,16,26^ However, these values range widely and may be insufficient for differentiating between normal eyes and those with early glaucoma.^15^ For this reason, Kita et al.^15^ first proposed the G/T ratio as a diagnostic parameter. They suggested that outer retinal thicknesses would not change as mGCC becomes thinner in glaucomatous eyes, resulting in a decrease in the G/T ratio, and they obtained results supporting this.^15^ Their study, conducted in the Japanese population, demonstrated that G/T ratio yielded significantly higher AUROC values than peripapillary RNFL in glaucoma patients, indicating that G/T ratio was more valuable in the diagnosis of glaucoma.^15^ However, these results were contradicted by a subsequent study conducted by Hollö et al.^24^ in the European population. Hollö et al.^24^ reported that G/T ratio had a lower AUROC value than those of mean RNFL and GCC thickness. The researchers attributed this discrepancy to the different ethnicities of the study groups.^24,27^

In the present study, we evaluated the AUROC values of RNFL, GCC, total macular thickness, and G/T ratio for differentiating between healthy individuals and patients with ocular hypertension, preperimetric glaucoma, and early glaucoma. Similar to the study by Hollo et al.^24^, our analysis showed that G/T ratio had a lower AUROC value compared to mean RNFL, mGCC, and total retinal thickness for all groups.^24^ Although the literature may present contradictory results due to ethnic differences, the G/T ratio has no diagnostic importance in practice, both in Europeans and in the present study conducted in our center. Instead of this calculation, a more practical approach would be to monitor the ganglion cell layer and peripapillary RNFL in glaucoma patients using OCT.

### Study Limitations

As a cross-sectional study, the inability to make the detailed distinction between preperimetric glaucoma and non-progressive glaucomatous optic nerve changes was one of its limitations, as in the study by Hollö et al.^24^ Prospective studies are needed to clearly make this distinction.

## Conclusion

In conclusion, the present study demonstrated that G/T ratio does not contribute significantly to the differentiation of patients with ocular hypertension, preperimetric glaucoma, and early glaucoma from the healthy population and has a lower diagnostic value compared to RNFL thickness, mGCC thickness, and total macular retinal thickness, as has been previously shown in various groups in the literature.

## Figures and Tables

**Table 1:**
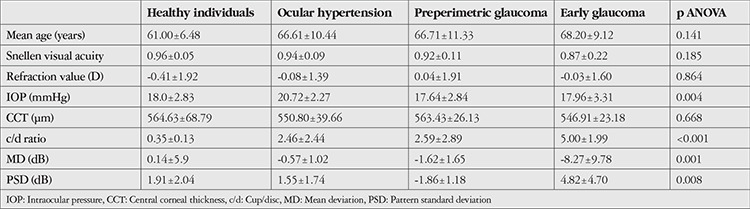
Clinical characteristics of the patients: mean age, visual acuity, mean refraction values, cup-to-disc ratio, intraocular pressure, central corneal thickness, and visual field values

**Table 2:**
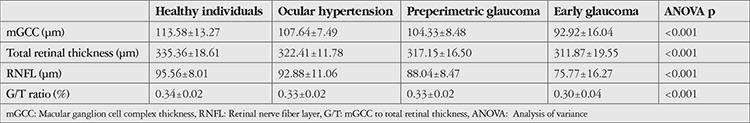
Comparison of mean mGCC, RSLT thickness, total macular retinal thickness, and G/T ratio values between the patient group

**Table 3:**
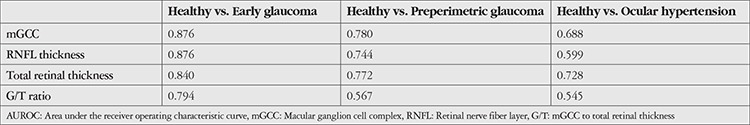
Area under the receiving operator characteristic curve (AUROC) values for mGCC, RSLT thickness, total macular retinal thickness, and G/T ratior
